# Comparison of Detailed and Simplified Models of Human Atrial Myocytes to Recapitulate Patient Specific Properties

**DOI:** 10.1371/journal.pcbi.1005060

**Published:** 2016-08-05

**Authors:** Daniel M. Lombardo, Flavio H. Fenton, Sanjiv M. Narayan, Wouter-Jan Rappel

**Affiliations:** 1 Department of Physics, University of California, San Diego, La Jolla, California, United States of America; 2 School of Physics, Georgia Tech University, Atlanta, Georgia, United States of America; 3 Department of Medicine, Stanford University, Palo Alto, California, United States of America; George Mason University, UNITED STATES

## Abstract

Computer studies are often used to study mechanisms of cardiac arrhythmias, including atrial fibrillation (AF). A crucial component in these studies is the electrophysiological model that describes the membrane potential of myocytes. The models vary from detailed, describing numerous ion channels, to simplified, grouping ionic channels into a minimal set of variables. The parameters of these models, however, are determined across different experiments in varied species. Furthermore, a single set of parameters may not describe variations across patients, and models have rarely been shown to recapitulate critical features of AF in a given patient. In this study we develop physiologically accurate computational human atrial models by fitting parameters of a detailed and of a simplified model to clinical data for five patients undergoing ablation therapy. Parameters were simultaneously fitted to action potential (AP) morphology, action potential duration (APD) restitution and conduction velocity (CV) restitution curves in these patients. For both models, our fitting procedure generated parameter sets that accurately reproduced clinical data, but differed markedly from published sets and between patients, emphasizing the need for patient-specific adjustment. Both models produced two-dimensional spiral wave dynamics for that were similar for each patient. These results show that simplified, computationally efficient models are an attractive choice for simulations of human atrial electrophysiology in spatially extended domains. This study motivates the development and validation of patient-specific model-based mechanistic studies to target therapy.

## Author Summary

Simulations generated by computers are often an effective way to study the dynamics of cardiac cells. A crucial component in these studies is the mathematical model that describes the electrical signal across the cells. The models vary from detailed, with numerous components, to simplified, with a minimal set of variables. While the detailed models contain more information, they are slower computationally. In this study we develop physiologically accurate computational human atrial models by fitting parameters of a detailed and of a simplified model to clinical data for five human patients. For both models, our fitting procedure generated parameter sets that accurately reproduced clinical data, but differed markedly from published sets and between patients, emphasizing the need for patient-specific adjustment. Both models were also capable of producing two-dimensional spiral wave dynamics for each patient. While the spiral waves differed significantly between patients, the models produced similar results for each case. These results show that simplified, computationally efficient models are an attractive choice for simulations of human atrial electrophysiology. This study motivates the development and validation of patient-specific model-based studies to target therapy.

## Introduction

Atrial fibrillation (AF) is the most common sustained cardiac arrhythmia and is associated with increased morbidity and mortality from stroke and heart failure [1]. Unfortunately, therapy for this condition is suboptimal due to its mechanistic complexity [2, 3]. Because of difficulties in studying AF mechanisms in humans, and since animal models of AF may differ from human AF, mechanistic studies of arrhythmias are increasingly turning to computational modeling to bridge the gap between clinical unmet needs and cellular studies.

Essential in these computational studies is the choice of the electrophysiological model which simulates the membrane potential through a set of parameterized equations that describe the ion channels. This model can range in complexity from detailed [4–8], which describe as many channels as possible, to simplified [9–11], which capture only essential dynamical features of cardiac tissue. However, these computational models are rarely validated in humans, and their parameters are typically based on imprecise, incomplete, or animal data.

We set out to address this problem by developing computational models that recapitulate cellular and tissue behavior in human AF. We used three sets of clinically obtained data from the left atria in 5 different patients with clinical AF at invasive electrophysiological studies. The data included action potential (AP) morphology, excluding the upstroke due to pacing artifacts, AP duration (APD), and conduction velocity (CV) restitution curves obtained during controlled pacing using a monophasic action potential (MAP) catheter, and maps of AF propagation obtained from direct contact wide-area multipolar basket catheters [12]. We used a simulated annealing fitting procedure to adjust the model parameters such that the numerical results fit the AP morphology and the restitution curves simultaneously. This was done first for the detailed Koivumäki *et al* (KKT) atrial myocyte model [7] which extends earlier detailed models [5, 6] to account for Ca^2+^ dynamics in the sarcoplasmic reticulum. We show that our fitting procedure was able to generate parameter sets that accurately reproduce clinical data. These sets differ significantly between patients and are markedly different than the published one. We then fitted the clinical restitution curves and the full AP morphology obtained from the KKT model (i.e., including the upstroke) to the simpler Fenton-Karma (FK) model [9]. We show that we are able to obtain parameters that can reproduce the clinical data and the fitted AP morphology well. Of note, a modeling sequence in which the FK model is fitted to clinical data, followed by a KKT fit to the FK output is equally possible.

Finally, we simulated spiral wave reentry in two-dimensional sheets using the fitted parameters sets for both models in all patients. We find that the dynamics of the spiral waves are similar for each patient. This suggests that simplified, computationally efficient models can be used to investigate spatio-temporal dynamics of cardiac activation. Our study shows how numerical models can be tailored to patient-specific clinical data, an important step towards guiding therapy based on individual AF mechanisms.

## Methods

Detailed Methods are available in Supporting Information.

### Clinical mapping

We acquired data from 5 patients with atrial fibrillation undergoing ablation for standard clinical indications, of age 64.2±10.6 years, left atrial diameter 42±3 mm and left ventricular ejection fraction 60±10%. All patients were studied after discontinuing all anti-arrhythmic medications for over 5 half-lives (amiodarone in 1 patient was discontinued 1 year earlier). MAPs were recorded during incremental pacing from slow heart rates to AF onset[13]. In brief, a deflectable 7F MAP catheter (EP Technologies, Sunnyvale, CA) was advanced to record AP in the antra of the right and left superior pulmonary veins. The protocol was completed before ablation. Patients in AF were electrically cardioverted to yield sinus rhythm, and the protocol started 10 minutes later. APs were recorded from distal poles of the MAP catheter while pacing the proximal poles. The close proximity of the recording and pacing poles necessitated special treatment for the first 30 ms of each AP morphology, as detailed in the Supporting Information. The resulting AP morphology captures repolarization but does not include the upstroke. We paced for >84 beats at each cycle length (CL) of 500 (baseline), 450, 400, 350, and 300 ms, then in 10 ms steps to AF or capture failure, whichever came first. Further details regarding signal processing, APD, and how activation time data was used to determine CV can be found in the Supporting Information.

### Numerical simulations

Simulations were carried out using the monodomain equation:

$$\frac{du}{dt}=D\nabla^{2}u-\frac{I_{ion}}{C_{m}}$$

where *u* is the membrane voltage, *C*_*m*_ represents the membrane capacitance, D is the diffusion coefficient (1D) or isotropic tensor (2D) and *I*_*ion*_ represents the membrane currents. Simulations in our fitting procedure were carried out in homogeneous 1D cables, consisting of 100 elements with a spatial discretization of 0.02 cm and a time step of 0.01 ms using no-flux boundary conditions. Decreasing the time step to 0.005 ms adjusted the CV restitution values by less than 4.5% for the FK model, and less than 1.5% for the KKT model while decreasing the spatial discretization to 0.01 cm changed these values by less than 7%. For the 2D simulations, we solved the monodomain equations in isotropic sheets of at least 9.6x9.6 cm using a square computational grid, again with a spatial discretization of 0.02 cm and a time step of 0.01 ms using an explicit Euler method. Spiral wave reentry was generated through cross-activation and spiral tip trajectories were computed using a previously published algorithm [9]. It is also important to note that while all simulations were carried out with isotropic tissue, actual tissue is heterogeneous. Including tissue anisotropy would require more detailed data on tissue conduction and fiber orientation.

Computations were performed using the C++ language and MPI parallelization on a high-performance workstation consisting of dual quad-core Xeon E5-2637 CPUs. Typical fitting simulations starting with the published parameters as initial conditions consisted of 50 iterations and required approximately 6 CPU hours for the FK model and 32 hours for the KKT model. This time can be significantly reduced if the initial parameter values are close to the final results. For instance, if two patients have similar dynamics, then the fitted parameters for one can be used as the starting point for the other. 2D simulations were performed on a GPU parallel computing platform with a Nvidia Tesla K40 graphics card. Computing 1000 ms of spiral wave propagation on a 512x512 grid required approximately 8 min for the FK model and 37 min for the KKT model.

### Electrophysiological models

In this study, we employed a version of the FK model which consists of four variables, three gating variables and the membrane potential, and 24 parameters [14]. Three of these parameters were fixed (see Supporting Information), resulting in 21 adjustable parameters. The KKT model consists of 43 variables and more than 100 parameters [5, 7] and, as described in the Supporting Information, fits were carried out by allowing 21 parameters to vary (S1 Table). When shifted 50% away from their original published values, each of these parameters was found to increase the error by 1 to 65%. Here, error is quantified as specified in the Supporting Information. Many of these parameters were shifted by even larger amounts in the final fits.

### Curve-fitting procedure

We used a simulated annealing fitting procedure in which parameter values are repeatedly adjusted in an attempt to minimize error functions which compare the numerical results to the clinical data set (see S1 Text). Unlike other algorithms, simulated annealing samples a large region of parameter space and does not automatically reject parameter choices that do not improve the fit [15]. This is done by assigning an artificial “temperature” which determines the amount of variation of parameters for each iteration and is slowly reduced during the fitting procedure [15]. Note that this temperature is a variable of the fitting procedure, and is not related to any physiological value in the models or data. This algorithm has been successfully applied to biological data [16, 17]. Simulations were started from a high temperature [18] and typically consisted of 50 iterations after each of which we reduced the temperature by 10%.

## Results

### Clinical data

**Fig 1:**
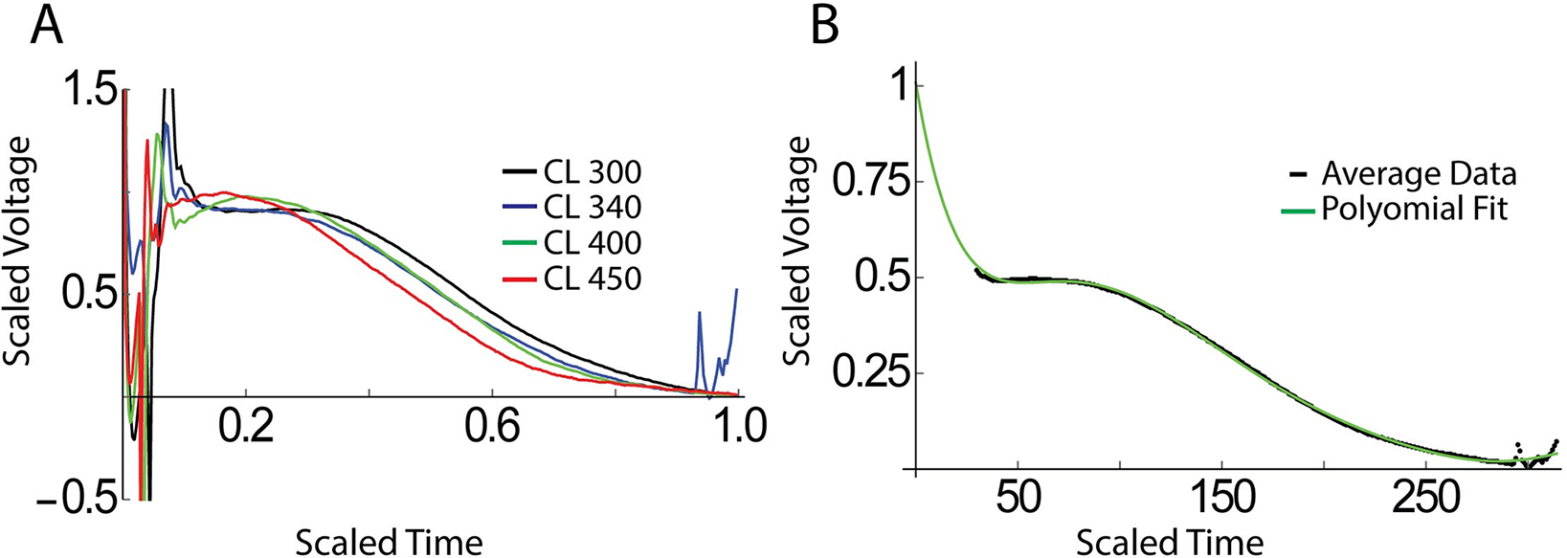
Determination of AP shape from clinical data. (A) MAP data in one of our patients (2) for different CLs (in ms). Time is rescaled and runs from 0 (stimulus) to 1 (repolarization). (B) Clinical data, corrected for pacing artifacts averaged over all CLs, shown as symbols and the corresponding polynomial fit.

In Fig 1A we show the clinically obtained AP morphologies for different CLs in one of the five patients, with voltage rescaled to span 0 and 1 and APD rescaled to 1 (raw APD90 ranged from 223–313 ms). Importantly, AP shapes are roughly independent of the CL, allowing us to define an average AP morphology for all CLs. This morphology, after the first 10 ms due the pacing artifacts has been removed, is shown in Fig 1B, together with its high-order polynomial fit (solid line). The resulting smooth AP morphology can then be used in the fitting procedure and can be adjusted to the required APD by a simple time dilation or contraction.

**Fig 2:**
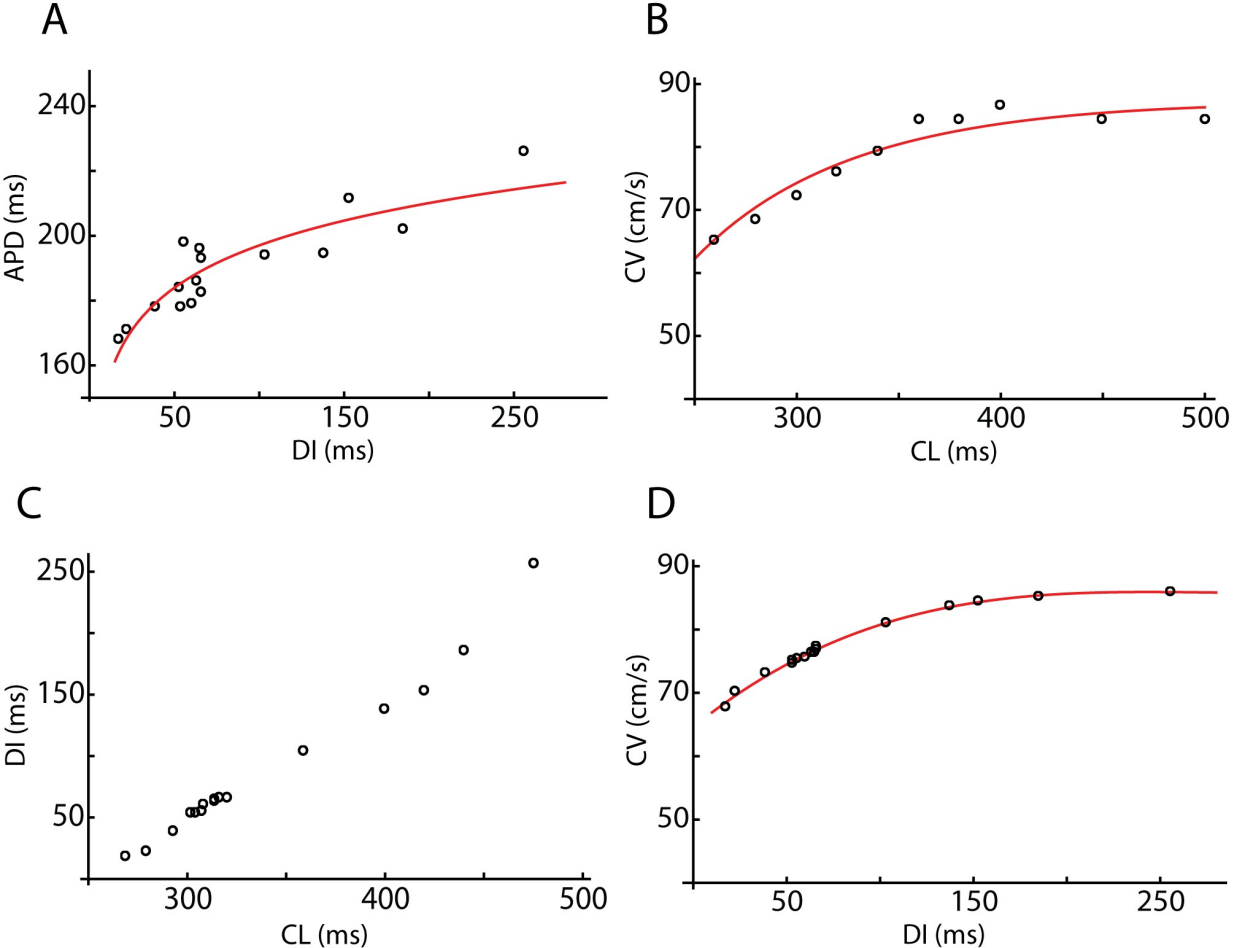
Clinical restitution data for patient 3. (A). The APD as a function of DI as determined from the MAP electrode (symbols) and the polynomial fit to the data (solid line). (B) CV as a function of CL using the activation times of the basket electrodes. (C) DI as a function of CL from the MAP data. (D) CV restitution curve computed using the data in (B) and (C). The symbols correspond to the values of the DI in (B).

Fig 2 shows examples of APD and CV restitution curves used in our fitting study for one of our patients (#3). Fig 2A shows APD restitution as a function of diastolic interval (DI) from the MAP data (symbols) along with a logarithmic fit (S1 Text). The clinical data relating CV and CL for the same patient, together with the polynomial fit, is shown in Fig 2B while in Fig 2C we have plotted the DI as a function of CL. The CV restitution curve based on the polynomial fit in Fig 2B and the dependence of DI on CL shown in Fig 2C is plotted in Fig 2D. The CV data for the remaining patients are shown in S1 Fig and S2 Fig.

### Parameter fits

As discussed above, the clinical data does not incorporate information about the AP upstroke. This upstroke, however, is largely responsible for the wave front dynamics and a meaningful comparison of spatio-temporal dynamics between the results of the KKT and the FK model is only possible if the upstroke in both models is similar. To enable such a comparison, we chose to first fit the clinical data to the KKT model. The resulting AP morphology, now including the upstroke, and clinical restitution curves were then used as fitting input to the FK model. This fitting sequence ensures that, in the case of a successful fit, the AP upstroke in both models is similar. We have verified that switching the order of the fitting procedure did not change the computational times in a significant manner.

**Fig 3:**
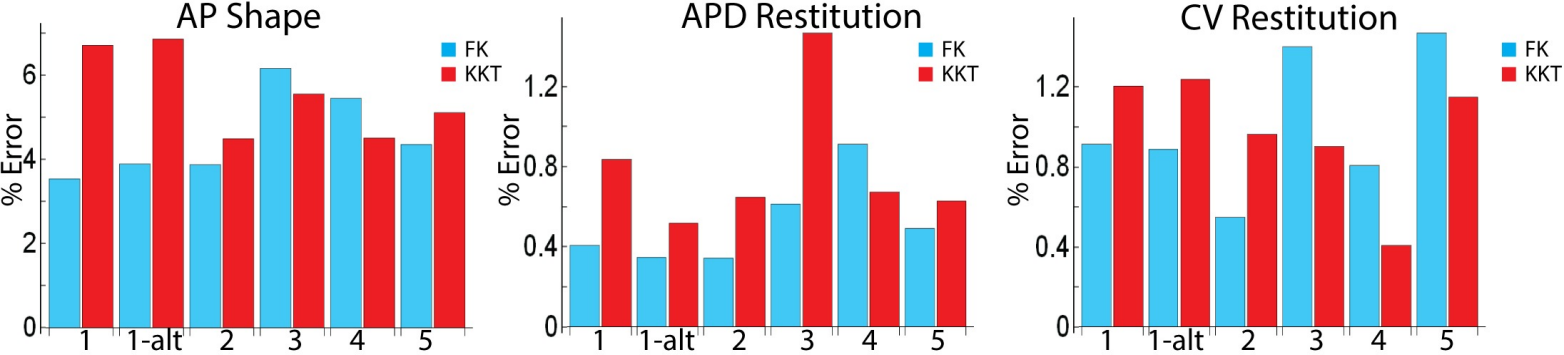
Error estimates of the obtained fits. The average error in both models is shown for the morphology, APD, and CV restitution curves for all patients, including the alternate parameter set for patient 1.

For the KKT model, we fitted the parameters to the clinical data of the 5 patients. The resulting values of the five parameter sets are listed in S2 Table. The parameters for the FK model, fitted to the KKT output, can be found in S3 Table. For one of the patients, #1, we determined a second, alternate, set of parameters, by using different initial conditions for the parameter values. We quantified the accuracy of our fits by determining the average error for each fitted point (see Methods). A full summary of the results is shown in Fig 3, and discussed in more detail below.

For reference, the percent error for the original published parameters of each model was also quantified. The average percent error in AP shape ranged from 100% to 226% for the FK model, and from 10% to 24% for the KKT model. The APD restitution ranged from 26% to 37% for the FK model, and 4.5% to 39% for the KKT model. The CV restitution ranged from 28% to 57% for the FK model, and 40% to 63% for the KKT model. We note that the original parameter set for the FK model was not chosen to describe atrial myocytes.

### Action Potential morphology

**Fig 4:**
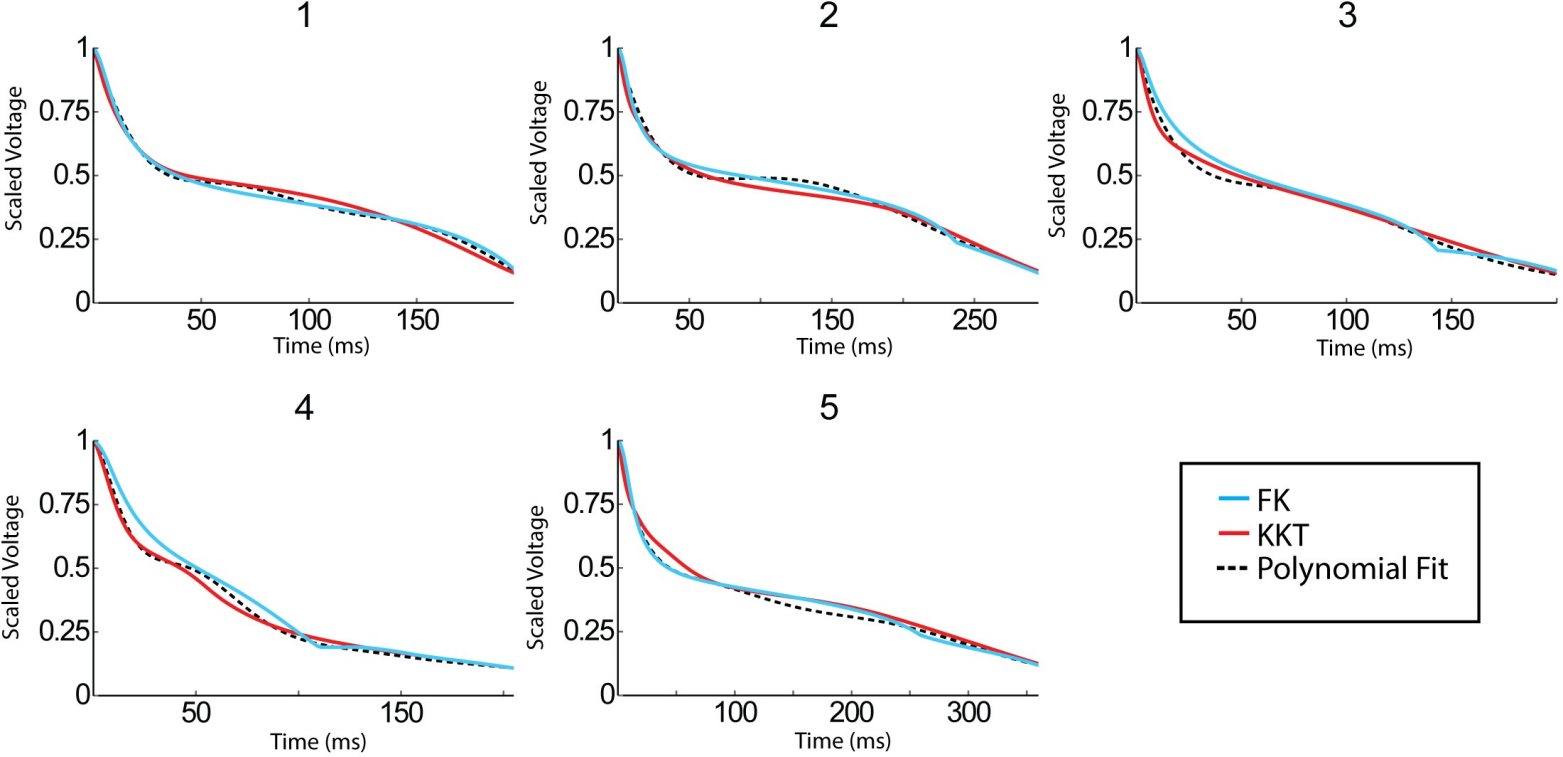
Clinical AP morphology compared to model AP morphologies obtained by fitting the model parameters. The average clinical AP morphology, corresponding to the largest DI value, is shown as a dashed line while the FK model is shown in blue and the KKT model is in red.

Fig 4 shows the AP morphology, corresponding to the largest DI value, obtained in all patients as dashed lines. Even though AP morphologies are similar, they differed in their precise shapes. The resulting morphologies from the KKT model, using parameters obtained from the fitting procedure, are shown in red. In this Figure, we show the shapes corresponding to the largest S2 stimulus (which differs for all patients) that was applied during the S1-S2 pacing protocol. Also shown in Fig 4, in blue, are the morphologies obtained by the FK model, fitted to the AP shapes from the KKT model.

A visual inspection reveals that both models can accurately reproduce the AP morphology for these patients. We quantified the accuracy of our fits by determining the average error for each fitted point (see Methods), and found that both models have a total error less than 6.8% when averaged over all S2s (Fig 3). The largest average error for a single S2 in the FK model was 11% (patient 4) while the largest error in the KKT model was 8% (patient 1). Furthermore, as shown in S3 Fig, the upstroke of the AP is similar between the KKT and FK models.

### APD restitution curves

**Fig 5:**
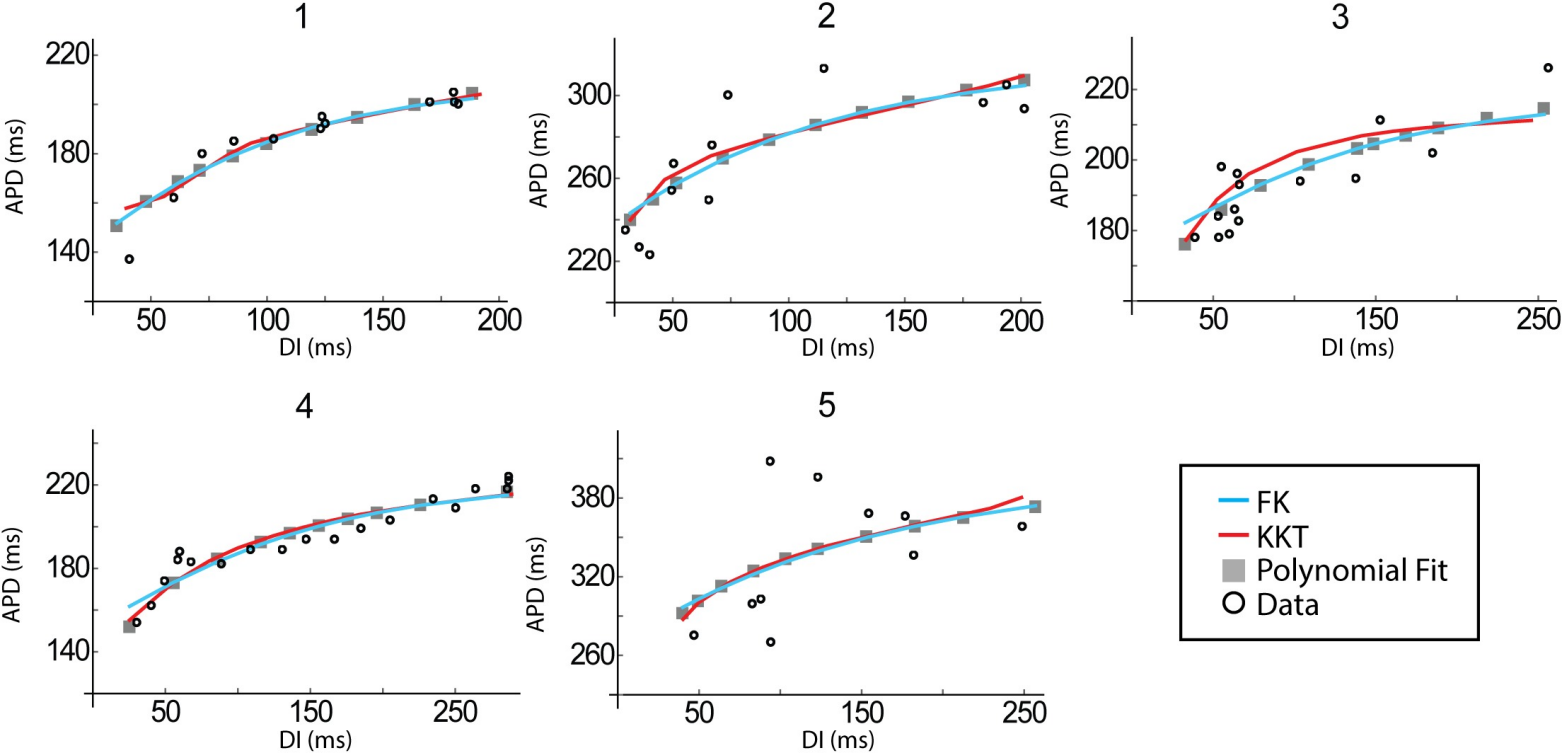
Model APD curves can be fitted to clinical data. The clinically determined APD and its polynomial fit are shown as open and closed symbols, respectively, for the 5 patients. The results from the fitting procedure is shown in blue (FK) and red (KKT).

Fig 5 displays the polynomial fits to the clinically obtained APD restitution curves as gray symbols. Note that while the polynomial is a continuous function, only data points used for the model fits are shown. As expected, the general shape of this data is identical, with decreasing APD for decreasing DI [19]. The most noticeable difference between patients is the change in maximum APD values. While patient 1 only reaches to approximately 220 ms, the maximum APD of patient 5 is close to 380 ms. Furthermore, the maximum slope of the restitution curve was also different from patient to patient, ranging from 0.57 for patient 3 to 1.15 for patient 2.

Both models (blue and red lines) are shown to reproduce the gray symbols well and overlap for most of the DI range, demonstrating that the model parameters can be adjusted to reproduce a range of restitution curves. One noticeable difference between the two models is that for our fitted parameter sets the KKT model tends to have a larger slope than the FK model for DI less than 50ms. As for the AP morphology, we can quantify the percent error of the model fits (Fig 3). We found that for the KKT model the average error over the entire APD restitution curve was less than 1.5% for all patients. For the FK model, the average error was below 1% for all patients. Thus, there was little difference in the error between the FK and KKT models.

### CV restitution curves

**Fig 6:**
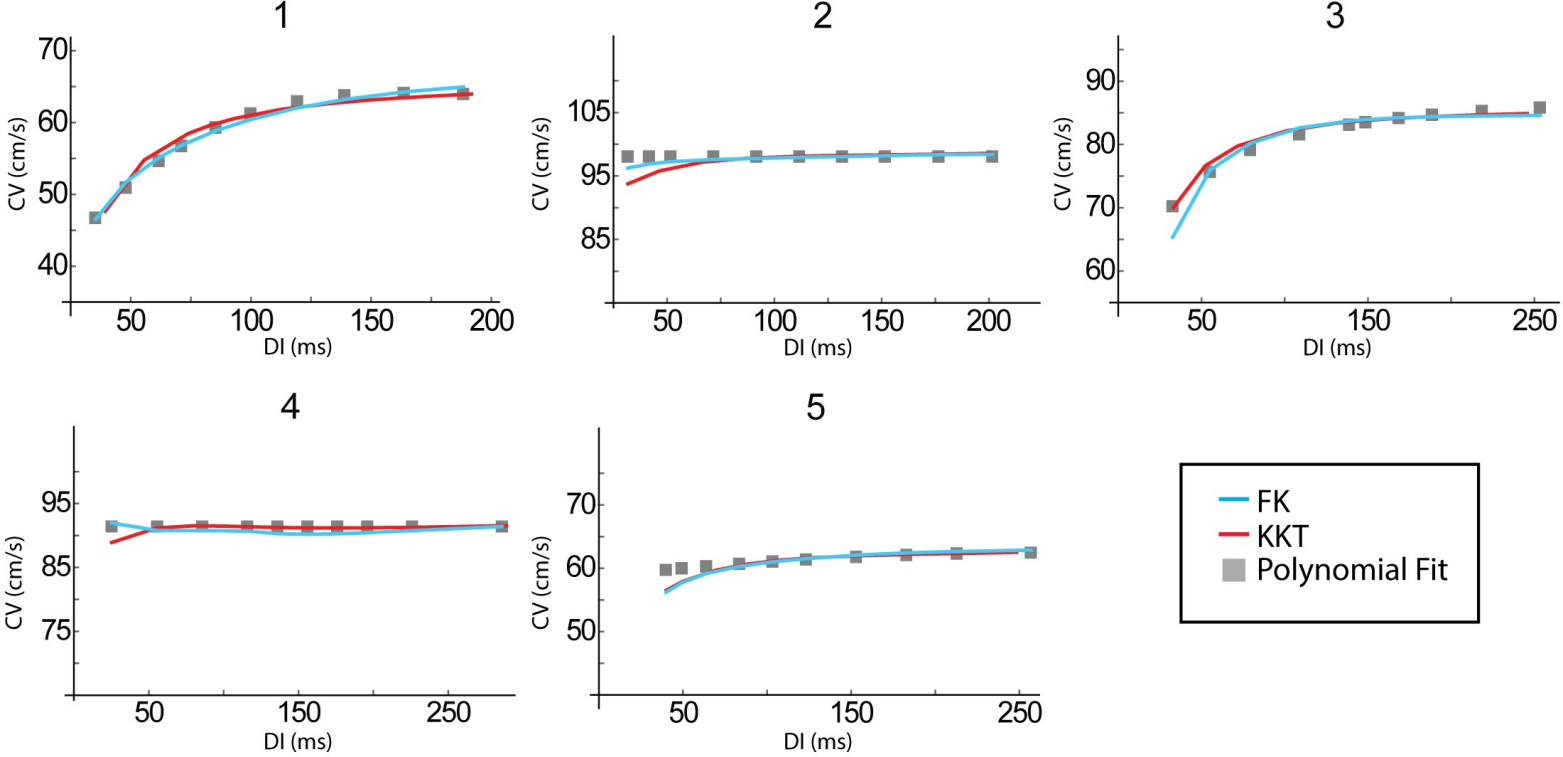
The conduction velocity as a function of DI for each of the 5 patients. No raw data was available for the CV restitution curves, though the points calculated from the inverse activation time data can be seen in Fig 2. Only the polynomial fit to these is shown here, along with the model curves.

The polynomial fits to the clinical CV restitution data are shown in Fig 6 as gray symbols. For three patients (2, 4, and 5) we found that this restitution curve is approximately flat while for patients 1 and 3 it decreases as the DI decreases. This is consistent with earlier reports that found that CV restitution can be flat or can decrease for decreasing DI [20]. The fits from both models are shown as solid lines and can be seen to match the clinical data over the entire range of DIs. The average error for the KKT model was found to be less than 1.3% for all patients (Fig 3) while the largest single point error is for small DI in the patients with flat CV restitution where the fitted CV differs from the clinical CV by 5.3% (patient 5). The cumulative error in the FK model was less than 1.5% for all patients and the largest deviation from clinical CV is for small DI in patient 3 (6.9%).

### Spiral wave propagation

**Fig 7:**
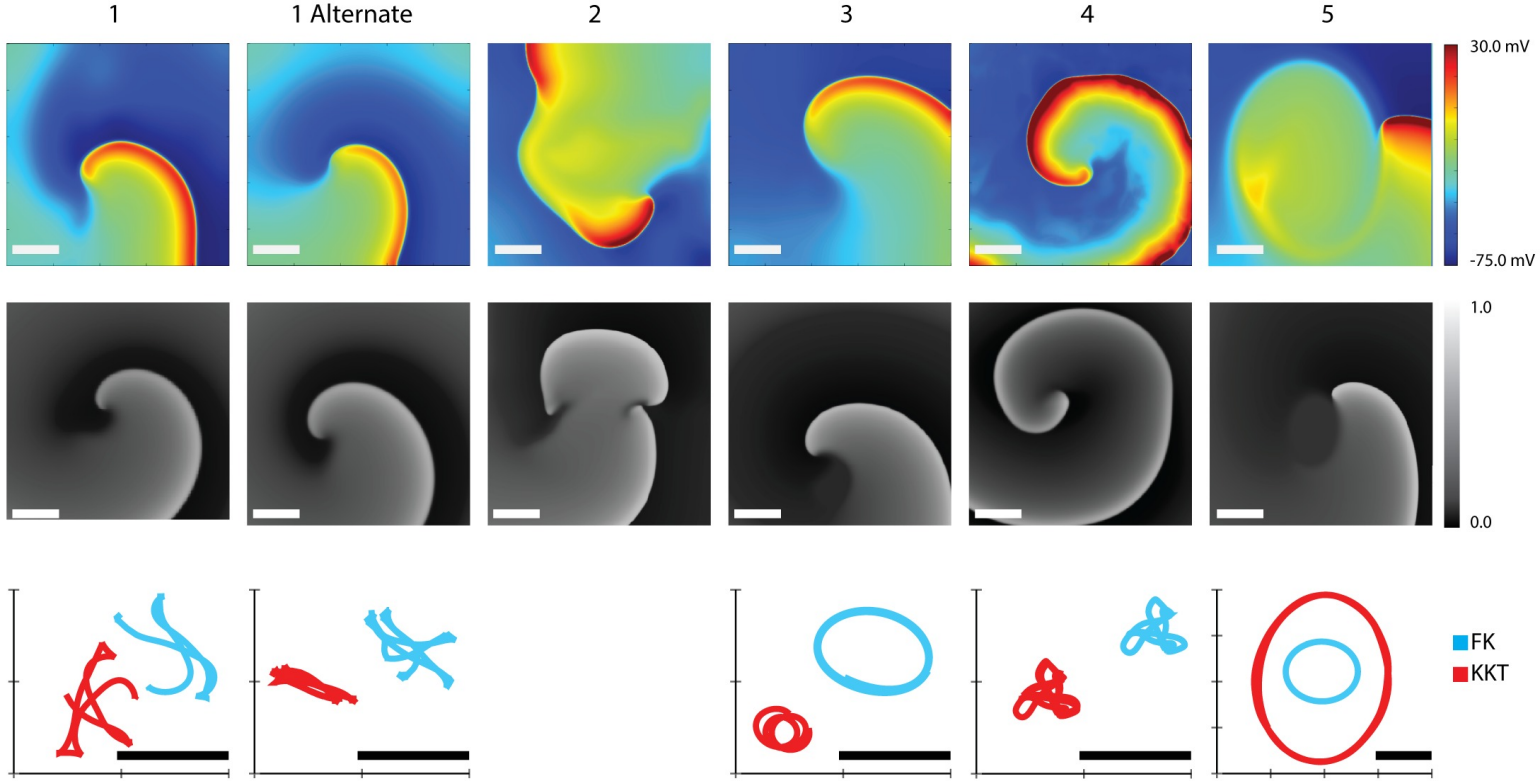
Spiral wave patterns in 2D for the fitted parameters sets. The top row shows snapshots of the activation pattern following the initiation of a spiral wave in the KKT model using the fitted parameter set for each patient. The membrane voltage is shown using a color scale. The middle row show snapshots of the activation patterns obtained using the FK model with white (black) corresponding to depolarized (repolarized) tissue. In the bottom row we have plotted the corresponding tip trajectories of the KKT model (red) and FK model (blue). Note that the trajectory location is arbitrary, and that some spirals were spatially translated to facilitate comparison between the models. Scalebar: 2 cm.

Two-dimensional spiral waves were generated for each of the fitted parameter sets in both models. Snapshots of the resulting activation pattern for each patient, including the alternate parameter set for patient 1, are shown in Fig 7 where the membrane voltage in the KKT model is shown in a color scale and in the FK model in a gray scale with white (black) corresponding to high (low) voltage.

Both models produced stable spiral waves in 4 out of 5 patients, with patient 2 exhibiting spiral wave breakup. In the lower row of Fig 7 we have plotted the trajectory of the spiral tips of the stable spirals (red for the KKT model and blue for the FK model). The trajectories in the two models show a similar pattern for all patients, including the alternative set. The scale of the meander pattern is very similar for patient 1 and 4 while slightly different for patients 3 and 5. The rotation period in the KKT model ranged from 125 ms (patient 4) to 457 ms (patient 5). Spiral wave periods in the FK model were roughly similar, differing from 2% (patient 2; 196 ms for the KKT model and 200 ms for the FK model) to approximately 20% (patient 5; 457 ms vs. 370 ms).

## Discussion

The present study performed detailed analyses of the ability of 2 computational models for atrial tissue, the detailed KKT model and the simpler FK model, to recapitulate APD and CV dynamics and AP morphology in a series of carefully studied patients with clinical AF. We found that the parameters of both the simplified and detailed models can be adjusted to reproduce clinically observed tissue behavior. We also found that these parameters varied significantly from patient to patient and from published parameter sets, indicating the need for personalized model building. Furthermore, we simulated the spiral wave reentry using the model equations parameterized by our fits. The spiral wave dynamics are qualitatively similar for each patient between models. The fact that simplified models produce results that can accurately fit clinical data, can generate similar spatio-temporal dynamics that is similar to the dynamics from detailed models, and have a small computational cost suggests that they might be better suited to model cardiac arrhythmias in spatially extended domains than computationally expensive detailed models.

The current study is in several ways distinct from previous studies which attempted to fit computational models to data [21–25]. First, we used clinical data instead of animal or numerical data to modify the parameters of two computational models. Detailed AP and CV restitution data were obtained from the left atrium of 5 patients with clinical AF at electrophysiological study. Second, our fitting procedure was designed to fit not only temporal dynamics using single cell characteristics but also conduction velocity data, a measure of spatio-temporal dynamics. This was achieved by fitting simultaneously the AP morphology as well as APD and CV restitution curves and adjusting model parameters so as to minimize the difference between clinically determined and numerically obtained tissue characteristics. These tissue characteristics are widely considered to be essential features of cardiac tissue and were obtained using recording electrodes, thus representing measurements at discrete locations within the atrium. Thus, and in contrast to fitting schemes that attempt to fit in a sequential fashion, our final parameter set produces a morphology and restitution curves that are optimal fits to the entire clinical data set.

Our results indicate that we are able to fit the clinical data equally well with the FK model and the KKT model. Both models produce fits that vary less than 7% for the AP morphology and approximately 1% for the APD and CV restitution curves. In our fitting algorithm we allowed parameter values to be increased or decreased by an order of magnitude in the KKT model and allowed a variable range in the FK model. These ranges can clearly be easily adjusted, for example using experimentally obtained restrictions of permissible values. We find that at least several parameters vary significantly (> 2-fold) from patient to patient and that every parameter varies significantly in at least one patient (S2 and S3 Tables). In addition, we found that there exist multiple parameter sets that fit the clinical data equally well. This was determined by re-computing the best fit using different initial parameter values for patient 1 (S2 and S3 Tables). The errors from the two different parameter set are roughly equivalent (Fig 3) indicating that both sets have an equal goodness of fit.

In the FK model we allowed nearly all parameters (21) to vary while in the KKT model 21 parameters were fitted with many more held constant (S1 Table). These constant parameters were chosen based on their minimal effect on the AP morphology or because they are well-established (for example, the cell size and volume). Not restricting the available parameter space by fixing these values will render the fitting procedure computationally unfeasible. It should be noted, however, that modifying which parameters can be varied is straightforward. Furthermore, we have explicitly verified that the inclusion of 11 more parameters did not result in an improvement in the fit (see S4 Table).

Our results demonstrate that there can be multiple parameter sets with equal goodness of fit. This is perhaps not surprising given the large dimensionality of the parameter space which can lead to multiple local minima [24, 25]. Even reducing this dimensionality, however, does not necessarily guarantee a unique set of parameters that fit a specific data set. For example, we have explicitly verified that reducing the number of fit parameters in the KKT model to 5 (g_Ks_, g_K1_, g_Nab_, g_Cab_ and P_Na_) can still lead to multiple parameter sets that fit the AP morphology and APD restitution within less than 1% error. For this, we created four trial parameter sets by randomly varying the parameters within ±50% of their original values. On average, each of the new, and distinct, parameter sets found by our fitting algorithm varied by 17.0%, 10.0%, 8.7%, and 13.6% from the original values (S5 Table). Thus, even a reduced number of parameters can have multiple local minima in the goodness of fitness space. Of course, these sets produced identical AP morphology and APD restitution, but other model properties, including 2D or 3D activation dynamics, might differ.

It is perhaps also not surprising that the parameter sets determined for the detailed KKT model differ from the published ones. The model parameters appear in the explicit descriptions of ion channels which take the form of coupled differential equations. However the structure of these equations and their parameters are derived not only from human ion channel or whole cell data but also from animal cells [26], that may limit their general applicability to human modeling. In addition, the parameters are not always precisely determined, not all channels might be incorporated into the model, and some data is obtained at unphysiological temperatures. Indeed, several recent detailed comparative studies of detailed models has shown that they can exhibit dynamical behavior that is not consistent with cardiac tissue [27–29].

The fact that our current study produced parameter sets that widely varied from patient to patient points to the need of algorithms that can adjust parameters based on clinical data. This is important, as human atrial tissue is not homogeneous and different atrial cell types display different AP morphologies [30]. In addition, pathophysiological remodeling of atrial tissue is likely to be a heterogeneous process [31] and requires reformulating the channel parameters [32]. Thus, it is unlikely that a single set of parameters or perhaps even a single model is able to capture the behavior of the entire atrium, and the parameters will need to be adjusted.

We used the results from our fits to simulate spiral wave dynamics in both the FK and KKT model. This is highly relevant for AF since recent mapping studies have revealed that spiral wave reentry plays a central role in AF [12, 33] and that the position of these spiral waves are a promising target for localized ablation [34]. To better understand and address the role of reentry in cardiac tissue and the best way to ablate, simulations of ionic models in spatially extended domains with the characteristics of a particular patient are thus desirable.

To facilitate this comparison we ensured that the upstroke of the AP was similar in both models. With a similar AP morphology and restitution curves, the two models produced spiral wave with very similar dynamics, including stability of the spiral wave, tip trajectory and the spiral wave period. The slight differences between the two models are likely due to electrotonic effects and memory effects [35] that act at timescales that are longer than single stimuli [36].

The comparison between the two models suggests that simplified models might be more advantageous to use in simulations of spatially extended phenomena than detailed models. Clearly, simulating detailed models is computationally expensive, as it requires solving a large set of stiff differential equations and to date, only a limited number of computational studies have been carried out using detailed models in 2 [37] and 3D [38–40]. The FK model, and similar simple models, on the other hand, are computationally much more efficient than detailed models [29, 41], and have been extensively used to model cardiac dynamics of single cells and in 2D [42] and 3D geometries [14, 43]. This comes, of course, at the expense of detailed knowledge about the precise role of the channels in normal and abnormal cardiac rhythms. Nevertheless, as we have shown here, the parameters of simplified models can be adjusted to represent clinical data with equal precision compared to detailed models. Thus, detailed models might not represent cardiac tissue dynamics more precisely than simplified models, making the latter attractive choices for simulations of phenomena that do not depend on specific ion channels or if computational speed is critical.

Our fitting procedure is not limited to the data sets we used here and other clinically relevant data can be incorporated. For example, it is possible to include data about the onset of alternans, implicated in the initiation of AF [13, 44], without altering the fitting scheme. On the other hand, data that is inherently spatially extensive, for example spiral wave rotation periods, would require simulating activation fronts in 2D. If combined with AP morphology and restitution curve data, it should be possible to use that data to fit model parameters in a simultaneous fashion. Of note, that type of data requires high resolution mapping of activation fronts and is currently unavailable in humans. In addition, modifications in our fitting procedure in which, for example, the AP morphology counts more than the restitution curves are straightforward to implement.

It should also be clear that our methodology can be applied to other cardiac tissue, including the right atrium and the ventricles. Furthermore, it can be used to fit data obtained in different parts of the atrium, thus generating model parameters that are adjusted in a regional fashion. The electrophysiological models that are then created can be combined with detailed information about the atrial geometry to create truly patient-specific atrial models [45–47]. This might be particularly interesting when applied to diseased tissue [31] and could be combined with detailed mapping of structural tissue remodeling to create patient-specific models. These computational models might become an important tool in the study of cardiac arrhythmias, possibly resulting in a deeper mechanistic understanding of AF and the development of novel therapies.

Our study has several limitations. First, CV was estimated from the activation times of electrodes on the same spline. A more accurate determination would require constructing high-resolution isochrones in patient-specific geometries. Also, the AP shape was derived from MAP recordings, which are intrinsically noisy. Furthermore, these MAP recordings only represent local tissue characteristics and quantifying atrial heterogeneity would require multiple recording sites. In addition, since our MAP electrode was close to the stimulus location, we do not have accurate data for the upstroke part of the AP. Also, our spatially extended simulations are in homogeneous 2D sheets, thus ignoring tissue anisotropy and potential 3D effects. Finally, a quantitative comparison between our spatio-temporal simulations and clinical data is currently challenging and would require accurate patient-specific data on the dynamics of wave fronts. Such a comparison necessitates extending our simulations to include anisotropy and 3D properties and would require detailed data on tissue conduction and atrial geometry, along with highly-optimized fitting algorithms and is the subject of future research.

## Supporting Information

S1 Text Detailed Methods. Further information on the data acquisition, computational models, and the curve fitting procedure.
(PDF)

S1 Fig Raw CV data for the remaining patients. Shown is the CV as a function of CL for patients 1, 2, 4, and 5. The equivalent curve for patient 3 is shown in Fig 2B.
(PDF)

S2 Fig CV restitution curves for the remaining patients. Shown is the CV as a function of DI for patients 1, 2, 4, and 5. The equivalent curve for patient 3 is shown in Fig 2D.
(PDF)

S3 Fig Upstroke of the FK and KKT models. The upstroke part of the APs obtained from the KKT fits are shown in red while the corresponding AP shapes of the FK model are shown in blue. The time interval Δt between 10% and 100% of the upstroke amplitudes of the KKT model was used as additional fit condition for the FK model. Data is shown for the largest DI value.
(PDF)

S1 Table Adjustable Parameters in the KKT model. (PDF)

S2 Table Parameter values of the KKT model obtained by fitting for all 5 patients. (PDF)

S3 Table Parameter values of the FK model obtained by fitting for all 5 patients. (PDF)

S4 Table Parameter values of the KKT model obtained by fitting 32 parameters to data from patient 1. (PDF)

S5 Table Parameter values of the KKT model obtained by fitting 5 parameters to simulated AP and APD data produced by the original data set. (PDF)
