# Supplementary material for: Comparison of Detailed and Simplified Models of Human Atrial Myocytes to Recapitulate Patient Specific Properties
Source: PLoS Comput Biol. 2016 Aug 5;12(8):e1005060. doi: 10.1371/journal.pcbi.1005060 (PMC4975409; doi:10.1371/journal.pcbi.1005060)
Supplement: S1 Text — Further information on the data acquisition, computational models, and the curve fitting procedure. (PDF) [file pcbi.1005060.s001.pdf]

S1 Text. Detailed Methods

Data acquisition from MAP catheter

The signal was processed by first filtering it between 0.05 to 500 Hz. An average AP curve was constructed by normalizing the AP shapes for all CLs (see Fig. 1). Our clinical data contains several AP shapes (between 4-9) for each CL. This can be used to compute a distribution of shapes, along with a standard deviation as a measure of variance between individual shapes. For example, for patient 2, we find that this standard deviation ranges from 1% to 10% while for patient 4 it ranges from 1% to 27%. We can compare this variance to the standard deviation as computed between all normalized AP shapes and the "canonical" AP shape. This computation yields a variance of roughly 2% to 10%, and 3% to 23% for patient 2 and 4, respectively, indicating that the degree of uncertainty per CL is similar to the degree of uncertainty for all CLs. Thus, it is justified to use a canonical AP shape.

Since the MAP electrode was close to the pacing electrode, the first 30 ms of each AP morphology was discarded and the value of the voltage at 30 ms was assumed to be 50% of the maximum upstroke, based on recording studies of isolated human atrial cells [1], while the potential at t=0 was set to the maximum upstroke value. The potential was rescaled to have values between 0 and 1 and was then fitted to a twelfth-order polynomial (see Fig. 1).

Action potential duration (APD) was determined using validated software [2] with manual verification. After assigning AP onset as time of maximal computed upstroke (dV/dt), we determined APD_{90} as the interval from AP onset to 90% voltage recovery from phase II. Diastolic interval (DI) spans the interval from APD_{90} of the previous beat to AP onset and for each patient the minimum and maximum DI values (DI_{min} and DI_{max}) were determined. The resulting APD restitution curve, relating DI and APD, was fitted using a logarithmic function of the form a0+a1*ln(DI).

CV restitution was assessed at multiple atrial sites by analyzing activation times at each of 64 electrodes of a basket electrode. A basket catheter (Constellation, Boston Scientific, Natick, MA) was inserted into the left atrium through a trans-septal sheath by experienced operators, taking care to ensure good contact [3]. Activation time was then determined at each site using semi-automated software based on the most negative dV/dt from the filtered isoelectric baseline between paced activations. The relative dependence of activation times on the CL, and thus the shape of the restitution curve, was determined by averaging the inverse activation time over all sites for each CL value. The maximum CV was computed from the difference in activation time between the electrode closest to the stimulus site, displaying the earliest activation after simulation, and its neighboring electrode along the same spline, located at a fixed distance of 4 mm. Finally, the CV restitution curve was obtained by using the CL vs. DI data and by fitting a function of the form $a0+a1/DI+a2/DI^2+a3/DI^3$ to the data points (see Fig. 2). This resulted in CV values for a range of DI (DI_{min} to DI_{max}).

Koivumaki *et al* model

The KKT model extends earlier details models [4, 5] to account for Ca^{2+} dynamics in the sarcoplasmic reticulum and consists of 43 variables and more than 100 parameters [4, 6]. Attempting to fit all parameters is computationally challenging and we therefore restricted the number of variable parameters. Specifically, all spatial dimensions, such as the length of the

cell, and radius and width of the bulk cytosol, were kept constant. In addition, the membrane capacitance, low and high phospholipid concentrations, phospholipid dissociation constants, and extracellular concentrations were fixed. Also, varying the parameters for the sodium-potassium exchanger current typically caused the model to produce unphysical results including oscillations and these parameters were held constant. Finally, we performed a sensitivity analysis using the parameter values of the original study. In this analysis, we decreased every parameter value in turn by 80% and determined the resulting change in the APD and AP shape of a single computational cell. Parameters that led to a total change that was less than 20% were held constant throughout our fitting procedure. Of the 13 parameters determined in this way, two were found to result in improved fitting results and were included into our group of variable parameters. The final result is a set of 21 parameters that were allowed to vary and which are specified in S1 Table. The initial values of these parameters were chosen to be the ones reported in the original study [6] and parameter values were allowed to change by a factor of 10 in either direction. After completing the fitting procedure with 21 parameters the fitting algorithm was repeated with the inclusion of 11 more parameters (BNa, K_{dBNa}, I_{NaKmax}, k_{NaKK}, k_{NakNa}, g_t, g_{sus}, g_{Kr}, g_{CaL}, g_{If}), but no improvement was found in the fit.

In order to decrease the computation time of the simulations, extensive look-up tables were used for the currents [7]. To better facilitate comparison of model and clinical AP morphologies, we rescaled the membrane potential so that its maximum value in the entire S1-S2 stimulus sequence corresponds to 1 and its minimum value to 0.

Fenton-Karma model

The FK model [8] replaces the detailed description of the numerous atrial ion channels with a minimal set of equations that can accurately describe key dynamical features of cardiac tissue. The model contains only 3 or 4 variables, together with a limited number of parameters that can be modified to reproduce AP shapes originating from detailed models [8, 9] and experiments [10]. In this study, we employed a version of the FK model which consists of four variables, three gating variables and the membrane potential, and 24 parameters [11]. The equations for the gating variables *v,w,* and *d* read:

$$\frac{dv}{dt} = \frac{(1-\Theta(u-u_{na}))(1-v)}{\big(1-\Theta(u-u_v)\big)t_{vm} + \Theta(u-u_v)t_{vmm}} - \frac{\Theta(u-u_{na})v}{t_{vp}}$$

$$\frac{dw}{dt} = \frac{(1-\Theta(u-w))(1-w)}{t_{wm}} - \frac{\Theta(u-u_w)w}{t_{wp}}$$

$$\frac{dd}{dt} = \left(\frac{1-\Theta(u-u_d)}{t_{sm}} + \frac{\Theta(u-u_d)}{t_{sp}}\right) * \left(\frac{1+\tanh(x_k(u-u_{csi}))}{2} - d\right)$$

where *v* and *w* represent the fast and slow gating variable, respectively. The third gating variable is added to the original version of the model [8] to improve the shapes of the AP and CV restitution curves [11]. These gating variables vary between zero and one, with zero being

completely closed and one being completely open, and result in a fast inward, slow inward, and slow outward current. These currents, along with the equation for the time constant t_{so}, are given by:

$$I_{fi} = -\frac{v * (u - u_{na}) * (u_m - u) * \Theta(u - u_{na})}{t_d}$$

$$I_{so} = \frac{(u - u_o)(1 - \Theta(u - u_c))}{t_o} + \frac{\Theta(u - u_c)}{t_{so}}$$

$$I_{si} = -\frac{w * d}{t_{si}}$$

$$tso = tso_a + \frac{1}{2}(tso_b - tso_a) * \big(1 + \tanh\big((u - u_{so})x_{tso}\big)\big)$$

The values of u_o, u_m, and u_{na} were set to 0.0, 1.0, and 0.23 respectively, while the values of the remaining 21 parameters were determined using our fitting procedure. As initial conditions for these parameters we used set 3 of Ref. [9], modified to the 4 variable version of the model. The threshold values for the voltage, along with u_{so} and u_{csi}, were constrained to vary between the minimum and maximum value of the membrane potential (0 and 1, respectively). The diffusion constant was allowed to vary between 10^{-5} and 0.1 cm^2/ms, but none of the fits were more than an order of magnitude away from 0.001 cm^2/ms. In order to incorporate the range of possible values given in the parameter sets of Ref. [9] all remaining parameters were allowed to vary over several orders of magnitude.

Curve-fitting procedure

Two points along the 1D cable, consisting of 100 elements, were used to compute the fit to clinical data. Specifically, we used element N=70 for the APD and AP morphology fits and used element N=30 and N=70 to calculate the CV. We have verified that these points were sufficiently far from the boundaries to minimize boundary effects. The cable was stimulated at one end using an S1/S2 protocol similar to the one used in the patients, where S1=500 ms was repeated 5 times before each S2 stimulus. Before each fitting iteration we stimulated the cable 30 times with CL=500 ms to ensure that the model had reached a steady state.

For each patient, 10 S2 values were chosen such that the resulting DI, determined as the time interval between the end of the preceding AP and the time of the S2 stimulus, falls into the clinical DI range (DI_{min} to DI_{max}). The numerical algorithm then produces simulation data for the AP morphology, and for the APD and CV restitution curves, which can be simultaneously fitted to clinical data.

Accuracy of the fit was defined through an error function which measures the discrepancy of the numerical results and the clinical data as follows:

$$E = \frac{1}{M}\sum_{i=1}^{M}\frac{\left|x_i^{sim} - x_i^{clin}\right|}{x_i^{clin}}$$

where x_i^{sim} and x_i^{clin} are the values of the simulation and clinical data points, respectively, and where *M* is the total points that are used in the error function. Here, we have chosen *M=30* such that, as detailed below, each clinical data set contributes 10 points. The task of the fitting procedure is to search for the parameter set **k** that minimizes *E*: $\mathbf{k} = \arg\min_{\mathbf{k}}(E)$.

AP morphology fits for the KKT model were calculated by choosing 10 evenly spaced points along the AP curve generated by the model and comparing them to the corresponding points of the polynomial function derived from clinical data. Since the MAP recordings did not capture the initial phase of the AP, we did not fit the AP upstroke and set the rescaled potential to 1 at t=0. This was performed for each of the 10 S2 stimuli and the error functions was averaged over all S2 values. Since the clinical AP morphology was largely independent of the S2 value (Fig. 1) we rescaled the clinical AP shape to adjust to the required APD value. For both the APD and CV restitution curves, we compared the numerical value of APD and CV to the one obtained from the polynomial fits of the clinical data. At the start of the fits, we chose S2 stimuli that were equally distributed in the clinical range. After manually inspecting the obtained fits, the DI values were adjusted to ensure better fits in parts of the restitution curves that were not optimally captured by the parameter sets. This typically meant a larger emphasis on the lower than on higher DI values.

The fitting procedure for the FK model incorporated the same conditions as described above but also used upstroke times obtained in the KKT fits. Specifically, for each S2 in the KKT morphologies we determined the time interval Δt between 10% and 100% of the upstroke amplitudes (S3 Fig). This time interval was then used as an additional condition for the FK fitting procedure and ensures similar upstroke morphologies in both models.

References

1. Workman AJ, Marshall GE, Rankin AC, Smith GL, Dempster J. Transient outward K+ current reduction prolongs action potentials and promotes afterdepolarisations: a dynamic-clamp study in human and rabbit cardiac atrial myocytes. The Journal of physiology. 2012;590(Pt 17):4289-305. doi: 10.1113/jphysiol.2012.235986. PubMed PMID: 22733660; PubMed Central PMCID: PMC3473286.
2. Franz MR, Kirchhof PF, Fabritz CL, Zabel M. Computer analysis of monophasic action potentials: manual validation and clinically pertinent applications. Pacing and clinical electrophysiology : PACE. 1995;18(9 Pt 1):1666-78. PubMed PMID: 7491310.
3. Narayan SM, Krummen DE, Shivkumar K, Clopton P, Rappel WJ, Miller JM. Treatment of atrial fibrillation by the ablation of localized sources: CONFIRM (Conventional Ablation for Atrial Fibrillation With or Without Focal Impulse and Rotor Modulation) trial. Journal of the American College of Cardiology. 2012;60(7):628-36. Epub 2012/07/24. doi: 10.1016/j.jacc.2012.05.022. PubMed PMID: 22818076; PubMed Central PMCID: PMC3416917.
4. Nygren A, Fiset C, Firek L, Clark JW, Lindblad DS, Clark RB, et al. Mathematical model of an adult human atrial cell: the role of K+ currents in repolarization. Circulation research. 1998;82(1):63-81. PubMed PMID: 9440706.
5. Maleckar MM, Greenstein JL, Trayanova NA, Giles WR. Mathematical simulations of ligand-gated and cell-type specific effects on the action potential of human atrium. Progress in biophysics and molecular biology. 2008;98(2-3):161-70. Epub 2009/02/03. doi: 10.1016/j.pbiomolbio.2009.01.010. PubMed PMID: 19186188; PubMed Central PMCID: PMC2836896.
6. Koivumäki JT, Korhonen T, Tavi P. Impact of sarcoplasmic reticulum calcium release on calcium dynamics and action potential morphology in human atrial myocytes: a computational study. PLoS computational biology. 2011;7(1):e1001067.
7. Clayton RH, Bernus O, Cherry EM, Dierckx H, Fenton FH, Mirabella L, et al. Models of cardiac tissue electrophysiology: progress, challenges and open questions. Progress in biophysics and molecular biology. 2011;104(1-3):22-48. Epub 2010/06/18. doi: 10.1016/j.pbiomolbio.2010.05.008. PubMed PMID: 20553746.
8. Fenton F, Karma A. Vortex dynamics in three-dimensional continuous myocardium with fiber rotation: Filament instability and fibrillation. Chaos. 1998;8(1):20-47. Epub 2003/06/05. doi: 10.1063/1.166311. PubMed PMID: 12779708.
9. Fenton FH, Cherry EM, Hastings HM, Evans SJ. Multiple mechanisms of spiral wave breakup in a model of cardiac electrical activity. Chaos. 2002;12(3):852-92. Epub 2003/06/05. doi: 10.1063/1.1504242. PubMed PMID: 12779613.
10. Cherry EM, Fenton FH. Visualization of spiral and scroll waves in simulated and experimental cardiac tissue. New J Phys. 2008;10:125016.
11. Cherry EM, Ehrlich JR, Nattel S, Fenton FH. Pulmonary vein reentry--properties and size matter: insights from a computational analysis. Heart Rhythm. 2007;4(12):1553-62. Epub 2007/12/11. doi: 10.1016/j.hrthm.2007.08.017. PubMed PMID: 18068635.
