# Supplementary material for: Comparison of Detailed and Simplified Models of Human Atrial Myocytes to Recapitulate Patient Specific Properties
Source: PLoS Comput Biol. 2016 Aug 5;12(8):e1005060. doi: 10.1371/journal.pcbi.1005060 (PMC4975409; doi:10.1371/journal.pcbi.1005060)

S1 Figure. Raw CV data for the remaining patients. Shown is the CV as a function of CL for patients 1, 2, 4, and 5. The equivalent curve for patient 3 is shown in Fig. 2B.

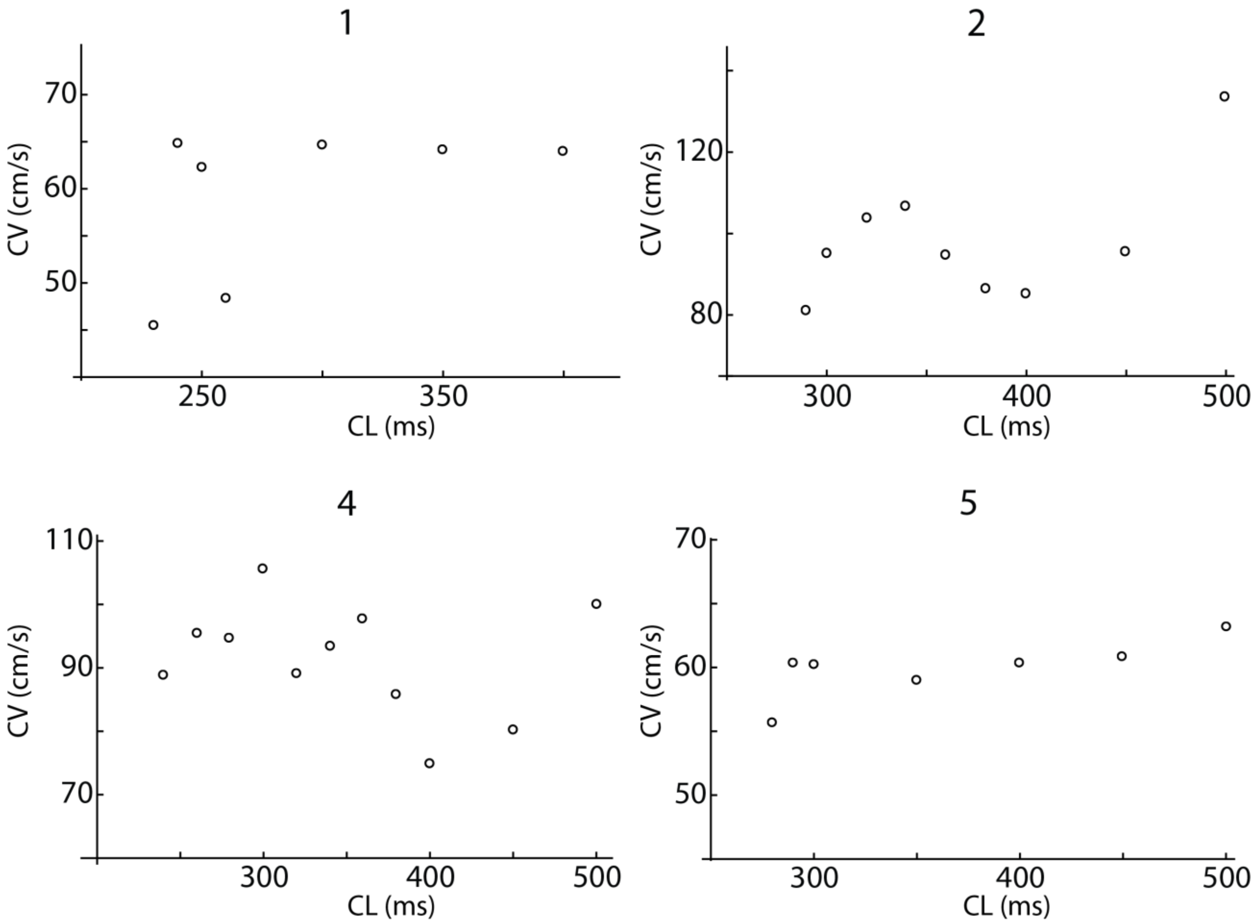

Supplement: S1 Fig — Shown is the CV as a function of CL for patients 1, 2, 4, and 5. The equivalent curve for patient 3 is shown in Fig 2B. (PDF) [file pcbi.1005060.s002.pdf]
