# Supplementary material for: Comparison of Detailed and Simplified Models of Human Atrial Myocytes to Recapitulate Patient Specific Properties
Source: PLoS Comput Biol. 2016 Aug 5;12(8):e1005060. doi: 10.1371/journal.pcbi.1005060 (PMC4975409; doi:10.1371/journal.pcbi.1005060)

S2 Figure. CV restitution curves for the remaining patients. Shown is the CV as a function of DI for patients 1, 2, 4, and 5. The equivalent curve for patient 3 is shown in Fig. 2D.

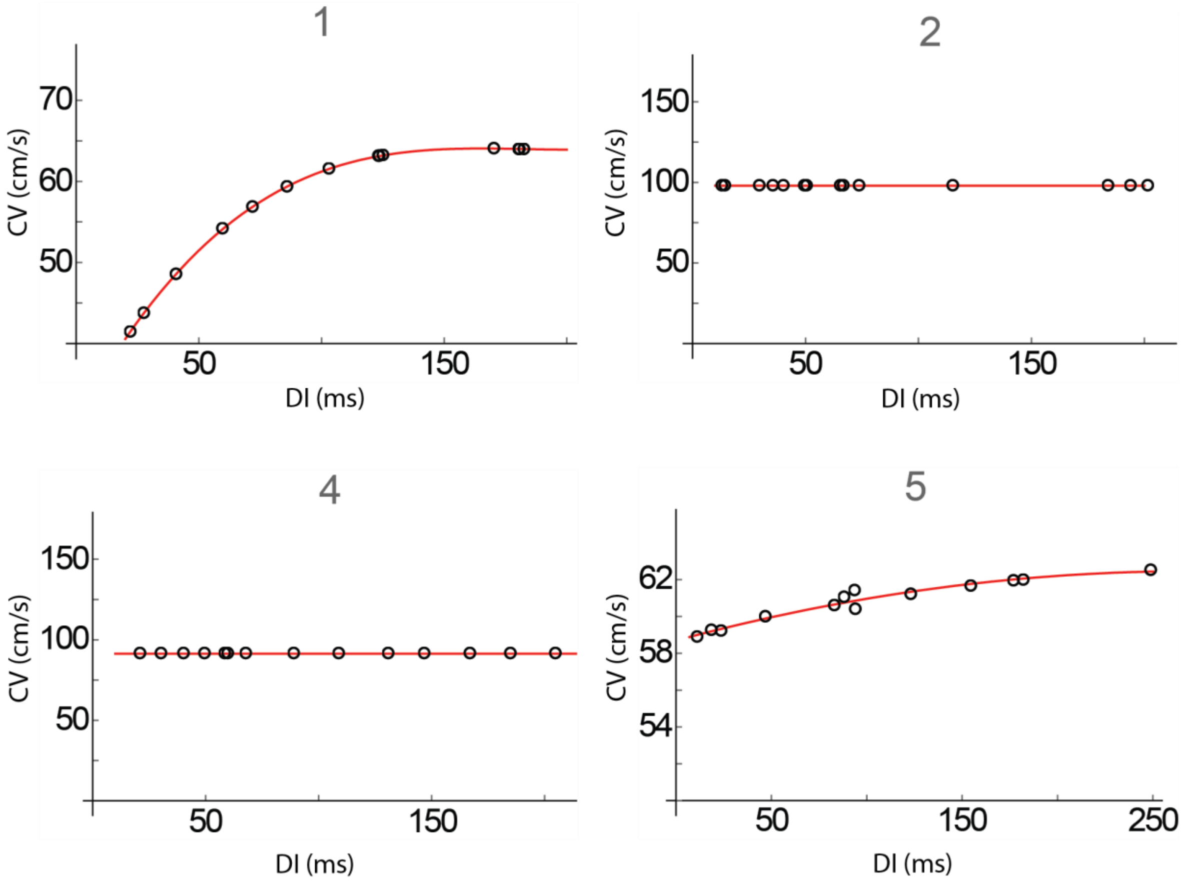

Supplement: S2 Fig — Shown is the CV as a function of DI for patients 1, 2, 4, and 5. The equivalent curve for patient 3 is shown in Fig 2D. (PDF) [file pcbi.1005060.s003.pdf]
