# Supplementary material for: Comparison of Detailed and Simplified Models of Human Atrial Myocytes to Recapitulate Patient Specific Properties
Source: PLoS Comput Biol. 2016 Aug 5;12(8):e1005060. doi: 10.1371/journal.pcbi.1005060 (PMC4975409; doi:10.1371/journal.pcbi.1005060)

S3 Figure. Upstroke of the FK and KKT models. The upstroke part of the APs obtained from the KKT fits are shown in red while the corresponding AP shapes of the FK model are shown in blue. The time interval Δt between 10% and 100% of the upstroke amplitudes of the KKT model was used as additional fit condition for the FK model. Data is shown for the largest DI value.

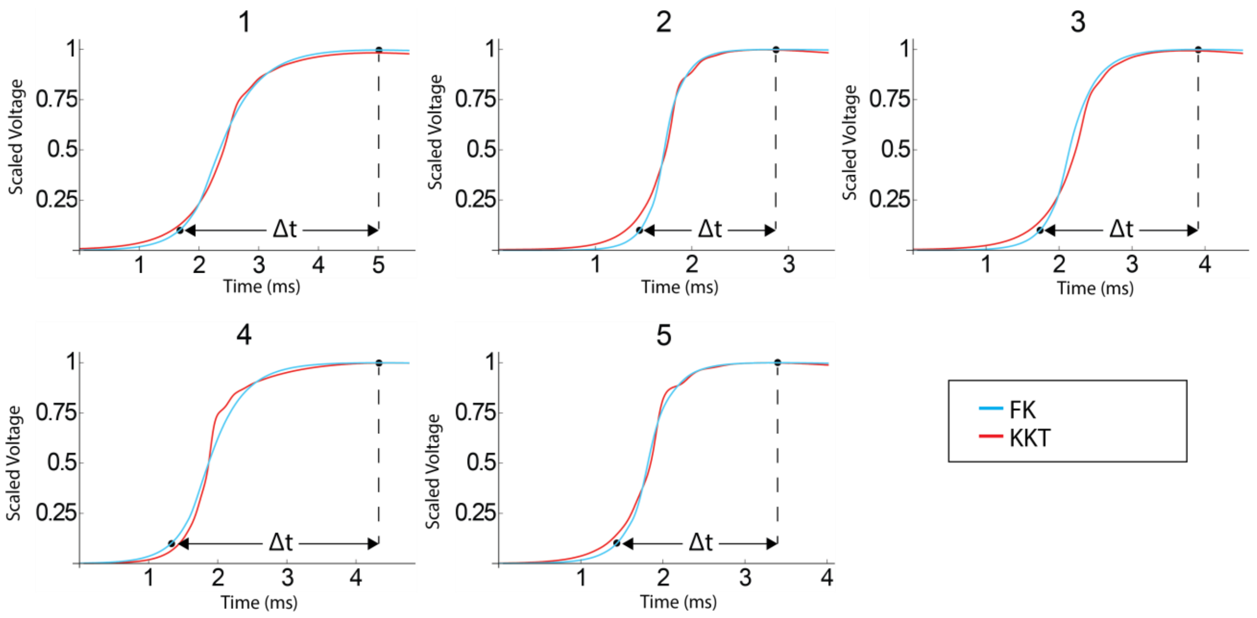

Supplement: S3 Fig — The upstroke part of the APs obtained from the KKT fits are shown in red while the corresponding AP shapes of the FK model are shown in blue. The time interval Δt between 10% and 100% of the upstroke amplitudes of the KKT model was used as additional fit condition for the FK model. Data is shown for the largest DI value. (PDF) [file pcbi.1005060.s004.pdf]
