# Supplementary material for: Comparison of Detailed and Simplified Models of Human Atrial Myocytes to Recapitulate Patient Specific Properties
Source: PLoS Comput Biol. 2016 Aug 5;12(8):e1005060. doi: 10.1371/journal.pcbi.1005060 (PMC4975409; doi:10.1371/journal.pcbi.1005060)
Supplement: S1 Table — (PDF) [file pcbi.1005060.s005.pdf]

S1 Table Adjustable Parameters in the KKT model.

BCa	Ca buffer concentration (mM)
K_{dBCa}	Dissociation constant (mM)
P_{Na}	Permeability of INa (nL/s)
E_{ca_app}	Apparent Reversal Potential for ICaL (mV)
kCan	Exponential factor in CaL time constant
k_{Ca}	Half max Ca in L-type current (mM)
g_{Ks}	conductance value for IKs (nS)
g_{K1}	conductance for K1 (nS)
g_{Nab}	conductance for background Na (nS)
g_{Cab}	conductance for background Ca (nS)
I_{CaPmax}	Max Ca pump current (pA)
k_{CaP}	Half max CA binding concentration (mM)
gamma	Position of energy barrier controlling voltage dependence of INaCa
d_{NaCa}	constant coefficient in INaCa $(mmol/L)^{-4}$
D_{Ca}	Ca diffusion coefficient ($\mu m^2/s$)
D_{CaSR}	Ca diffusion in SR ($\mu m^2/s$)
D_{CaBm}	Ca diffusion in buffer ($\mu m^2/s$)
D_{Na}	Na diffusion coefficient ($\mu m^2/s$)
k_4	pump rate from SERCA to SR (s^{-1})
k_{SRleak}	SR leak scaling parameter (s^{-1})
D (diffusion)	diffusion between cells (cm^2/ms)
