# Supplementary material for: Comparison of Detailed and Simplified Models of Human Atrial Myocytes to Recapitulate Patient Specific Properties
Source: PLoS Comput Biol. 2016 Aug 5;12(8):e1005060. doi: 10.1371/journal.pcbi.1005060 (PMC4975409; doi:10.1371/journal.pcbi.1005060)
Supplement: S2 Table — (PDF) [file pcbi.1005060.s006.pdf]

S2 Table Parameter values of the KKT model obtained by fitting for all 5 patients.

	1	1 Alt.	2	3	4	5	original
BCa	0.07488	8.113E-03	0.04279	0.07364	0.01963	0.02143	0.02400
KdBCa	9.716E-04	2.065E-03	3.393E-03	2.382E-03	2.166E-03	1.806E-03	2.380E-03
PNa	2.141E-03	2.370E-03	6.411E-03	2.920E-03	6.380E-03	4.580E-03	1.800E-03
Eca_app	91.26	45.95	60.86	46.92	286.5	75.24	60.00
kCan	1.051	0.8541	1.432	0.5913	1.085	1.100	2.000
kCa	3.848E-04	6.056E-04	1.328E-03	4.955E-04	2.088E-04	8.424E-04	6.000E-04
gKs	0.6346	0.3174	0.7901	0.7217	9.312	1.453	1.000
gK1	6.255	5.256	3.890	2.634	2.225	4.353	3.450
gNab	0.1224	0.1095	0.01034	0.05286	8.472E-03	0.08860	0.06060
gCab	0.09949	0.09934	0.06184	0.05006	0.01022	0.09369	0.09520
ICaPmax	3.365	0.7689	1.271	2.472	0.2239	4.219	2.000
kCaP	9.420E-04	2.133E-04	4.120E-03	1.248E-03	2.603E-03	8.411E-04	5.000E-04
gamma	0.5941	0.7096	0.4043	0.6359	0.04726	0.2300	0.4500
dNaCa	4.671E-05	1.127E-04	3.352E-05	1.291E-04	1.315E-04	8.072E-05	3.000E-04
DCa	166.4	1927	327.3	467.8	7076	871.6	780.0
DCaSR	54.30	6.212	98.19	78.69	41.34	59.84	44.00
DCaBm	71.55	38.25	88.32	49.71	131.3	29.72	25.00
DNa	0.4046	0.4908	0.07802	0.1427	0.1522	0.08442	0.1200
k4	6.813	119.5	6.806	5.587	10.38	3.880	13.00
kSRleak	3.073E-03	0.02863	2.632E-03	0.02296	0.04236	3.789E-03	6.000E-03
D	2.164E-03	1.945E-03	2.413E-03	2.785E-03	2.127E-03	1.209E-03	1.000E-03
