# Supplementary material for: Comparison of Detailed and Simplified Models of Human Atrial Myocytes to Recapitulate Patient Specific Properties
Source: PLoS Comput Biol. 2016 Aug 5;12(8):e1005060. doi: 10.1371/journal.pcbi.1005060 (PMC4975409; doi:10.1371/journal.pcbi.1005060)
Supplement: S3 Table — (PDF) [file pcbi.1005060.s007.pdf]

S3 Table Parameter values of the FK model obtained by fitting for all 5 patients.

	1	1 Alt.	2	3	4	5	original
uc	0.1313	0.2171	0.2579	0.2131	0.2069	0.2588	0.1300
uv	0.3085	0.1142	0.1799	0.1107	0.03489	0.1382	0.04000
uw	0.2635	0.2508	0.2566	0.2798	0.1788	0.2589	0.1300
ud	0.05766	0.1428	0.1943	0.1601	3.140E-04	0.1797	0.1300
tvm(ms)	57.12	46.77	40.31	35.75	971.3	45.15	19.60
tvp(ms)	2.189	1.759	1.349	1.247	2.243	2.194	3.330
twm(ms)	68.50	80.18	89.08	109.8	110.7	166.4	41.00
twp(ms)	871.4	749.5	777.0	751.8	616.0	836.3	870.0
tsp(ms)	1.110	1.484	1.144	1.487	16.29	1.315	1.000
tsm(ms)	1.7570	1.983	1.086	2.241	7.104E-03	0.764	1.000
ucsi	0.1995	0.2168	0.2722	0.2097	0.1682	0.2023	0.8500
xk	6.043	21.62	6.142	8.679	8.958	7.351	10.00
td(ms)	0.12990	0.08673	0.04456	0.06880	0.08511	0.06711	0.2500
to(ms)	15.17	17.05	23.45	18.31	6.754	18.28	12.50
tsoa(ms)	72.66	54.90	97.89	54.43	152.9	105.4	33.30
tsob(ms)	7.933	1.685	3.308	4.894	19.82	3.264	33.30
uso	0.4804	0.6520	0.4185	0.6804	6.013E-03	0.3497	0.8500
xtso	2.592	2.161	1.997	2.187	8.677	1.968	10.00
tsi(ms)	40.11	38.82	36.60	40.39	18.94	39.23	29.00
D(cm^2/ms)	1.611E-03	1.337E-03	1.405E-03	1.704E-03	2.696E-03	8.479E-04	1.000E-3
tvmm(ms)	1012	1321	1183	1187	120.5	1166	1250
