# Supplementary material for: Comparison of Detailed and Simplified Models of Human Atrial Myocytes to Recapitulate Patient Specific Properties
Source: PLoS Comput Biol. 2016 Aug 5;12(8):e1005060. doi: 10.1371/journal.pcbi.1005060 (PMC4975409; doi:10.1371/journal.pcbi.1005060)
Supplement: S4 Table — (PDF) [file pcbi.1005060.s008.pdf]

S4 Table Parameter values of the KKT model obtained by fitting 32 parameters to data from patient 1.

BCa	0.07825
KdBCa	9.926E-04
PNa	2.169E-03
Eca_app	89.83
kCan	1.051
kCa	3.810E-04
gKs	0.5992
gK1	6.334
gNab	0.1218
gCab	0.09771
ICaPmax	3.281
kCaP	9.287E-04
gamma	0.6117
dNaCa	4.497E-05
DCa	165.0
DCaSR	58.34
DCaBm	81.61
DNa	0.3983
k4	7.093
kSRleak	2.826E-03
D(diffusion)	2.149E-03
BNa	1.050
KdBNa	9.569
kNaCa	9.025E-03
INaKmax	71.28
kNaKK	0.9785
kNaKNa	11.26
gt	8.242
gsus	2.244
gKr	0.4762
gCaL	14.86
gIf	1.061
