# Supplementary material for: Comparison of Detailed and Simplified Models of Human Atrial Myocytes to Recapitulate Patient Specific Properties
Source: PLoS Comput Biol. 2016 Aug 5;12(8):e1005060. doi: 10.1371/journal.pcbi.1005060 (PMC4975409; doi:10.1371/journal.pcbi.1005060)
Supplement: S5 Table — (PDF) [file pcbi.1005060.s009.pdf]

S5 Table Parameter values of the KKT model obtained by fitting 5 parameters to simulated AP and APD data produced by the original data set.

	1	2	3	4	original
PNa	1.734E-03	1.778E-03	1.762E-03	1.780E-03	1.800E-03
gKs	0.5437	1.462	1.104	0.6031	1.000
gK1	3.614	3.401	3.543	3.410	3.450
gCab	0.09056	0.09498	0.09122	0.09897	0.09520
gNab	0.07642	0.06127	0.07521	0.04710	0.06060
